# Editorial: Brain-Computer Interfaces: Novel Applications and Interactive Technologies

**DOI:** 10.3389/fncom.2022.939202

**Published:** 2022-06-21

**Authors:** Jesus Minguillon, Ivan Volosyak, Christoph Guger, Michael Tangermann, Miguel Angel Lopez

**Affiliations:** ^1^Brain-Computer Interfaces Lab, Research Centre for Information and Communications Technologies (CITIC-UGR), Department of Signal Theory, Telematics and Communications, University of Granada, Granada, Spain; ^2^BCI Lab Kleve, Faculty of Technology and Bionics, Rhine-Waal University of Applied Sciences, Kleve, Germany; ^3^g.tec Medical Engineering GmbH, Schiedlberg, Austria; ^4^Artificial Intelligence Department, Donders Institute, Radboud University, Nijmegen, Netherlands

**Keywords:** Brain-Computer Interface, virtual reality, affective computing, human-machine interaction, biomedical signal processing, neuro-prostheses, wireless brain area networks, machine learning

A Brain-Computer Interface (BCI) allows people to communicate through brain signals without the need of any muscular movement. This modern technology may be used in assistive systems to enhance the communication capability of people with severe neuromuscular disorders such as Amyotrophic Lateral Sclerosis (ALS), locked-in syndrome (LIS), brainstem stroke, or spinal cord injury, cerebral palsy, muscular dystrophies, and multiple sclerosis. The recent integration of miniaturized electronics with wireless communication technologies has permitted the development of multimodal and wearable technologies for neural interfacing, as well as for the reproduction of real-and-virtual combination of environments and human-machine interactions (e.g., virtual and extended reality).

Nowadays, the combination of virtual and extended reality with brain-based technologies has facilitated the emergence of more sophisticated and effective neuro-rehabilitation therapies and assistive applications. For instance, virtual reality has been used for rehabilitation of cognitive and motor impairment in people with dementia by means of reminiscence therapies. Furthermore, recent work has demonstrated that the combination of conventional therapies with virtual reality and brain-based technologies for motor impairment rehabilitation can be more effective than conventional means alone. This exciting horizon of interactive brain-based technologies opens the door for new opportunistic applications in the fields of healthcare, telemedicine, assisted living, education, entertainment, culture, marketing and others.

This editorial presents the four research articles appearing in this Research Topic. These articles address different applications and challenges of BCI research such as objective psychological stress quantification, image decoding from the visual cortex and enhancement of BCI accuracy and response time. In these papers, non-invasive BCIs are combined with novel machine learning methods and other technologies such as virtual reality.

According to the American Psychological Association, more than 75% of adults report symptoms of psychological stress (resulting e.g. in headache or sleeping problems). In the last years many studies have addressed stress detection and quantification using different approaches. The combined use of bio-potentials [measured e.g. by means of electroencephalography (EEG) or electrocardiography (ECG)] and machine learning has proved its capability to accurately quantify and classify stress levels, outperforming classical approaches based on subjective report. In the paper “*Quantitative Assessment of Stress Through EEG During a Virtual Reality Stress-Relax Session*,” Perez-Valero et al. propose a method for quantitative stress assessment based on EEG spectral features and different regression algorithms. The authors conducted an experiment in which the participants underwent a stress-relax session while monitoring their EEG. The participants were stressed using the Montreal imaging stress task (MIST) and then relaxed during a virtual reality experience. The authors proved the stress assessment capability of their approach by comparing its output with the self-perceived stress level of the participants recorded using surveys. Using a random forest regression, they reported a mean squared percentage error (MSPE) of 10.62 ± 2.12 and a Pearson correlation coefficient (R^2^) of 0.92 ± 0.02.

Modern machine learning approaches allow extracting certain statistical patterns from EEG data e.g. for image reconstruction. The use of EEG overcomes the limitations of functional magnetic resonance imaging (fMRI) in terms of cost and availability. However, the low spatial resolution of EEG limits the performance of the proposed methods when reproducing aspects of the perceptual realism of an image (e.g., detailed shapes or sharp contours). Despite the low spatial resolution, texture images can be reconstructed from EEG since their representation in the visual cortex is based on global image statistics. In the paper “*Photorealistic Reconstruction of Visual Texture From EEG Signals*,” Wakita et al. propose a method for the reconstruction of texture images from visual evoked potentials recorded from humans viewing natural textures. This approach is based on a multimodal variational auto encoder (MVAE). The authors demonstrated the capability of the MVAE model to reconstruct images that perceptually look like the original textures with a photographic appearance.

Regarding BCIs, the limited performance of these systems hinders their consolidation as an effective assistive technology. Machine learning has been also employed to improve the performance of non-invasive BCI systems intended for typical applications such as two-dimensional cursor control. In the paper “*A Noninvasive BCI System for 2D Cursor Control Using a Spectral-Temporal Long Short-Term Memory Network*,” Pan et al. propose a framework based on a spectral-temporal long short-term memory (stLSTM) network for 2D cursor control. The authors reported outcomes in terms of control accuracy based on the root mean square error (RMSE) of the model's predicted velocities (an average RMSE reduction of 63.45% in comparison with the existing literature).

In addition to accuracy, the enhancement of the information transfer rate (ITR) is still one of the main challenges of BCI research. Most of the BCI systems reported in the literature provide ITRs that are not high enough for the intended application in realistic scenarios, what reduces their usefulness. The combined use of EEG and other techniques has been proposed to address the ITR challenge. In the paper “*Novel Hybrid Brain-Computer Interface for Virtual Reality Applications Using Steady-State Visual-Evoked Potential-Based Brain-Computer Interface and Electrooculogram-Based Eye Tracking for Increased Information Transfer Rate*,” Ha et al. propose the use of an electrooculogram (EOG)-based eye tracking to increase the ITR of a BCI in a virtual reality environment. In addition to eye activity, the proposed calibration-free hybrid (EEG and EOG) system uses steady-state visual evoked potentials (SSVEP). The authors reported that both the ITR and the accuracy of the hybrid system (i.e., SSVEP and EOG) were significantly higher than those of the non-hybrid system (i.e., SSVEP only) in a virtual reality environment.

## Author Contributions

All authors listed have made a substantial, direct, and intellectual contribution to the work and approved it for publication.

## Funding

JM was supported by Junta de Andalucia (Postdoctoral Fellowship Programme PAIDI 2020). ML was supported by Junta de Andalucia (Grant B-TIC-352-UGR20), the Spanish Ministry of Science, Innovation and Universities (Grant PGC2018-098813-B-C31) and the University of Granada (Grant PP2021.PP-28). IV was supported by German Federal Ministry of Education and Research (funding program Forschung an Fachhochschulen, contract number 13FH033EX0).

## Conflict of Interest

CG is the CEO of g.tec Medical Engineering GmbH. The remaining authors declare that the research was conducted in the absence of any commercial or financial relationships that could be construed as a potential conflict of interest.

## Publisher's Note

All claims expressed in this article are solely those of the authors and do not necessarily represent those of their affiliated organizations, or those of the publisher, the editors and the reviewers. Any product that may be evaluated in this article, or claim that may be made by its manufacturer, is not guaranteed or endorsed by the publisher.

